# The care manager as a key role for optimising resources/Las gestoras de casos como elementos claves en la optimización de recursos

**Published:** 2012-05-29

**Authors:** Mª Rosa García Cerdán, Mª Angels Fernández de la Fuente Pérez, Helen Fernández Pavón, Gemma Baldrich Berenguer, Alicia Solsona Serrano, Dolors Miguel Ruiz

**Affiliations:** Primary care (PC) nurse, Institut Català de la Salut (ICS), Barcelona, Spain; PC nurse, ICS, Barcelona, Spain; PC nurse, ICS, Barcelona, Spain; PC nurse, ICS, Barcelona, Spain; PC nurse, ICS, Barcelona, Spain; Nurse and Senior Lecturer at Sant Joan de Déu University School of Nursing, Barcelona, Spain

**Keywords:** continuity of care, resource rationalisation, resource optimisation, continuidad asistencial, racionalizar recursos, optimizar recursos

## Introduction

Continuity of care (CC) is based on patient and family care as the main element, with a bidirectional relationship between healthcare and social care services; the main aim being to avoid a lack of coordination between levels of care which could be detrimental to users [1].

CC is associated with patients perceiving a level of consistency and connectedness in their care experience over time. Three inter-related types of CC have been identified [2, 3]: a) relational: the perception of the user with regards to their relationship over time with one or more providers; b) informational: the perception of the user with regards the availability, use and interpretation of the information on previous events to provide care appropriate to their current circumstances, and c) management: the perception of the user that they receive the different services in a coordinated and complementary manner, without duplicity. Each type of CC is characterised by a series of dimensions and attributes and can be analysed in a specific clinical episode or from the general perceptions of users [1–3].

It has been reported that CC is associated with higher levels of user satisfaction [4–8], better perceived quality of life, greater use of preventive services [5–9], higher rates of adherence to treatment [7, 8] and decreases in hospital admission rates [5, 6, 8–10]. On the other hand, rapid technological advances and changes in the organisation of health services, as well as the growth in the prevalence of complex chronic diseases and the number of patients with multiple diseases, mean that patients tend to be seen by a large number of providers in different organisations and services. In turn, this makes it difficult to coordinate their care and, therefore, threatens CC [2, 11, 12]. For this reason, health systems usually include CC in their quality improvement plans for complex chronic diseases so that it is considered across the system in the ways of working and included in the training programmes of health professionals [13, 14].

In relation to this working model, the objectives of our case management service are:

1. To optimise and rationalise the use of services and of resources to prevent admissions due to clinical worsening of people with chronic diseases, that is, avoiding unnecessary transfers to hospital
2. To decrease visits to the emergency department by people with complex conditions, as well as their number of admissions and, if admitted, the length of their hospital stay
3. To empower family members by providing them with the necessary tools to avoid clinical worsening and manage risky situations.

## Description of the intervention

This intervention was based on the work carried out by a case management unit composed of nurses. Case Management is the process of systematic and dynamic collaboration to provide and coordinate health services for a given population. That is, it is a participative process to enable access to a range of options and services that cover patient needs, as well as reducing fragmentation of care and duplicity of services, while improving the quality of care and cost-effectiveness in the achievement of clinical targets [15].

Patients included on the list of the case management unit under study had complex conditions and had been referred from Primary Care (through review of patient clinical histories and referrals of health professionals) or Specialised Care (hospital admissions and social care services). A patient was considered to be complex on the basis of meeting 5 of the following 10 criteria, agreed by the case management unit:

1. ≥65 years old
2. Comorbidity according to the Charlson index [16]
3. Five or more drugs (including psychiatric drugs) taken continuously
4. Terminal stage of a disease
5. Barthel index [17] of ≤55, as an assessment of independence in the basic activities of daily living (BADL)
6. Dementia or cognitive deterioration (Pfeiffer’s Short Portable Mental Status Questionnaire [18] score >5)
7. Two or more unplanned hospital admissions due to clinical worsening in the previous 12 months
8. Three or more visits to a hospital emergency department in the previous 12 months
9. More than 2 falls in the previous 2 months
10. Living alone or with caregiver who can only provide a limited amount of support, as assessed by a social risk indicator (‘*TIRS*’ ≥1) (Annex 1)

A descriptive study was carried out comparing a period of one year (from January to December 2010) before and after the management unit was established. For this, data were collected on the basis of the 10 aforementioned criteria, agreed in the case management unit, indicating the level of complexity of the patients.

The geographical area, on the outskirts of the city of Barcelona, is the district of Baix Llobregat Litoral which, including the towns of Gavà and Viladecans among others, can be defined as urban and has a population of 120,000. The different levels of care are offered by the same health service provider. On the other hand, they do have different IT systems, but health professionals can access patient data through shared medical records (SMRs).

The study population was composed of 78 patients with complex chronic conditions. The inclusion criteria were: patients being registered in the unit and having a level of complexity ≥5 at baseline. After the overall assessment of the patients, the intervention carried out by the nurses was to agree treatment with the patient, producing a personalised care plan with the collaboration of informal caregivers and of the professionals involved in the patient’s care at the various levels of care.

Data were retrieved from the primary care and the shared medical records. They was then analysed using the most suitable statistical tests for each of the variables.

## Results

**Figure 1.**
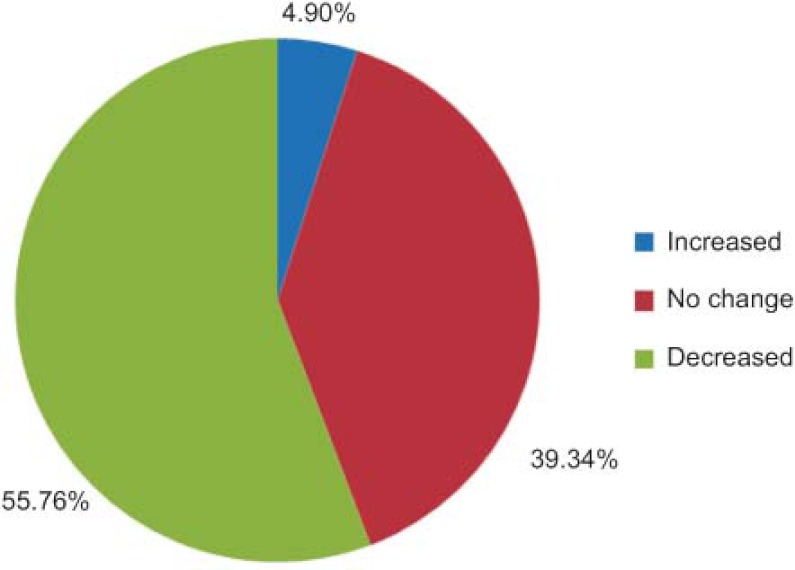
Level of complexity.

The level of complexity decreased in 55.76% of patients (n=44) (Figure 1).

We also observed a decrease in the number of visits to the emergency department and in the length of hospital stay, both results being statistically significant (Table 1).

## Conclusions

The case management model facilitates the three main types of continuity of care: relational, informational and management. The observed reduction in the complexity of the patients indicates that patient health problems were being more effectively monitored by nurses, which enabled worsening in their chronic conditions to be avoided, and in turn a more rational use of health service resources. This indicator is related to activities affecting the three aforementioned types of continuity of care with an associated improvement in patient perceived quality of life and level of satisfaction.

Further, our data demonstrate that the intervention significantly reduced Emergency Department attendances and hospital admissions, while inpatient stays tended to be shorter.

## Poster abstract Spanish

## Introducción

La continuidad asistencial (CA) está basada en la atención al paciente y núcleo familiar como eje principal, a través de una interrelación bidireccional entre servicios sanitarios y socio-sanitarios con un objetivo asistencial principal, evitar descoordinaciones entre niveles asistenciales que perjudican directamente al usuario [1].

La continuidad asistencial (CA) es el grado de coherencia y unión de las experiencias en la atención que percibe el paciente a lo largo del tiempo. Se distinguen tres tipos de CA [2, 3] interrelacionados entre sí: a) de relación: es la percepción del paciente sobre la relación que establece a lo largo del tiempo con uno o más proveedores; b) de información: es la percepción del usuario sobre la disponibilidad, utilización e interpretación de la información sobre acontecimientos anteriores para proporcionar una atención apropiada a sus circunstancias actuales, y c) de gestión: es la percepción del usuario de que recibe los diferentes servicios de manera coordinada, complementaria y sin duplicaciones. Cada tipo de CA se describe mediante una serie de dimensiones y atribuciones y puede ser analizado a partir de un episodio clínico concreto o a partir de las percepciones generales de los usuarios [1–3].

La CA se asocia a una mayor satisfacción de los usuarios [4–8], mejor calidad de vida percibida, mayor utilización de los servicios preventivos [5–9], mayor tasa de adherencia a los tratamientos [7, 8] y disminución del índice de hospitalizaciones [5, 6, 8–10]. Sin embargo, los rápidos avances tecnológicos, los cambios en la organización de los servicios de salud, el aumento de las enfermedades crónicas complejas y del número de pacientes pluripatológicos, hacen que los usuarios sean atendidos por un elevado número de proveedores en organizaciones y servicios distintos, hecho que dificulta la coordinación de la atención, y por tanto, amenaza la CA [2, 11, 12]. Por ello los sistemas sanitarios integran la CA en los distintos planes de calidad para enfermedades crónicas complejas de tal forma que se considere transversalmente en sus formas de trabajo y sea incluida dentro de los planes de formación de los profesionales [13, 14].

En consonancia con este modelo de trabajo, los objetivos de nuestro servicio de gestión de casos son:

1. Optimizar y racionalizar el consumo de servicios y la utilización de recursos para prevenir los ingresos por agudizaciones de las personas con patologías crónicas, evitando traslados innecesarios a los servicios hospitalarios.
2. Disminuir el número de ingresos, tiempo de hospitalización y visitas a urgencias en personas en situación de complejidad.
3. Capacitar a los componentes del núcleo familiar facilitando las herramientas necesarias para resolver situaciones de riesgo y evitar descompensaciones.

## Descripción de la experiencia

La experiencia parte del trabajo realizado en una unidad de gestión de casos formada por enfermeras. Se entiende por gestión de casos el proceso de colaboración sistemático y dinámico para proveer y coordinar servicios sanitarios en una población determinada; es decir, un proceso participativo para facilitar opciones y servicios que cubran las necesidades del paciente, a la vez que reduce la fragmentación y duplicación de servicios y mejora la calidad y la relación coste-efectividad de los resultados clínicos [15].

La cartera de pacientes de la unidad de gestión de casos estaba compuesta por pacientes complejos derivados de Centros de Atención Primaria (revisión historias clínicas y derivaciones de los profesionales sanitarios) y Atención Especializada (hospitalización y atención socio-sanitaria). El perfil de paciente complejo se definió en base al cumplimiento de 5 de los 10 criterios consensuados por la unidad de gestión de casos y descritos a continuación:

1. ≥65 años.
2. Comorbilidad en base al índice de Charlson [16]
3. 5 o más fármacos o psicofármacos de forma continua.
4. Proceso terminal
5. Valoración de las actividades básicas de la vida diaria (ABVD) a partir de un índice de Barthel ≤55 o menos [17]
6. Demencia o deterioro cognitivo (Test Cognitivo de Pfeiffer >5) [18]
7. 2 o más ingresos no planificados en el hospital por exacerbación en los últimos 12 meses.
8. 3 o más visitas a urgencias hospitalarias en los últimos 12 meses.
9. más de dos caídas en los últimos dos meses
10. Vivir solo o con cuidadores con capacidad limitada de soporte. Indicador de riesgo social (TIRS≥1) (Anexo 1)

Se realizó un estudio descriptivo, comparativo de antes y después, durante el periodo de enero a diciembre de 2010, mediante la recogida de datos, en base a los criterios de complejidad consensuados en la unidad de gestión de casos (10 criterios descritos anteriormente) que nos informaban del grado de complejidad de los pacientes.

El área geográfica, de 120.000 habitantes, está situada en el Baix Llobregat Litoral, extrarradio de Barcelona, con población urbana, que abarca los municipios de Gavà y Viladecans. Los diferentes niveles de atención asistencial están gestionados por el mismo proveedor de servicios sanitarios. Cuentan con sistemas de información distintos, pero se puede acceder a los datos de los pacientes desde la Historia Clínica Compartida (HCC).

La población de estudio estuvo constituida por 78 pacientes en situación de patología crónica y compleja. Los criterios de inclusión fueron: pacientes incluidos en la unidad, con grado de complejidad inicial de ≥5. La intervención de las enfermeras tras valoración integral del paciente fue realizar un pacto terapéutico con el paciente elaborando un plan de cuidados individualizado en el que intervenían los profesionales implicados en la atención del paciente de los diferentes niveles asistenciales y los cuidadores informales.

La recogida de la información se hizo a través de la explotación de datos de la historia clínica de Atención Primaria e HCC. El análisis de datos se realizó con las pruebas estadísticas más adecuadas para cada tipo de variable.

## Resultados

**Figura 2.**
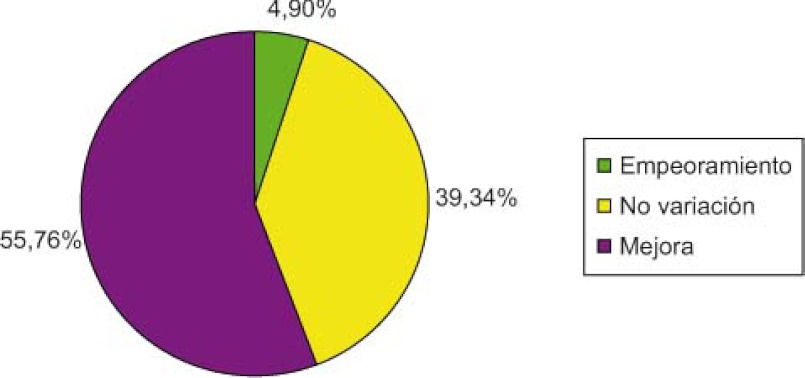
Grado complejidad.

El grado de complejidad mejoró en un 55,76% (n=44) de los pacientes (Figura 2).

Se observó una disminución de las visitas a urgencias y del número de ingresos hospitalarios, siendo estos resultados estadísticamente significativos (Tabla 2).

## Conclusiones

El modelo de gestión de casos facilita la continuidad asistencial en los tres ámbitos de actuación: relación, información y gestión. La mejora del grado de complejidad de los pacientes indica que el nivel de vigilancia de las enfermeras respecto a los problemas de salud de los pacientes es mayor, evitando así reagudizaciones de sus procesos crónicos, con lo que se racionaliza el consumo de servicios sanitarios. Este indicador se desprende de los tres ámbitos de actuación mencionados anteriormente donde hay una mejora en la percepción de calidad de vida y el grado de satisfacción por parte de los pacientes. Los resultados obtenidos demuestran una disminución en el número de ingresos hospitalarios significativo, de las visitas a urgencias, y en el tiempo de estancia hospitalaria.

## Figures and Tables

**Table 1.** Results before and after the intervention

	Before	After	p-Value
Visits to the emergency department	3.03 (2.80)	1.58 (1.77)	0.02
Number of admissions	2.34 (1.8)	1.27 (1.5)	<0.0001
Length of hospital stay in days	30.94 (21.8)	17.83 (17.14)	ns

**Tabla 2.** Resultados antes y después

	antes	después	significativas
Visitas urgencias	3,03 (2,80)	1,58 (1,77)	p=0,02
Número ingresos	2,34 (1,8)	1,27 (1,5)	<0,0001
Días de ingreso	30,94 (21,8)	17,83 (17,14)	ns

**Annex 1.** Social Risk Indicator

1. **Living alone or with family with little capacity to provide support:** an individual who lives on their own or with people who have some level of disability (for reasons of age, illness or impairment)		YES		NO
2. **Problematic family relationships:** where this refers to any type of family conflict (from disagreements to broken relationships)		YES		NO
3. **Family are not readily able to take on responsibility for caring for the patient:** where this refers to work commitments, other dependent relatives, exhaustion and other personal limitations.		YES		NO
4. **Unsatisfactory or poor personal hygiene:** as stated		YES		NO
5. **The accommodation does not meet patient needs:** where this refers to architectural barriers, lack of space, damp, lack of basic utilities (running water, electricity, etc.)		YES		NO
6. **An apparent lack of financial resources:** this refers to statements by the family and also the impression of professionals (by observation)		YES		NO

**Score:** ’Yes’ response to 1 or more item=Social risk.

**Anexo 1.** Indicador de riesgo social

1. **Persona que vive sola o familia con capacidad limitada de soporte:** persona que no convive con nadie o que convive con personas con algún tipo de discapacidad (por motivos de edad, de enfermedad o de disminución)	SI	NO
2. **Persona con relación familiar conflictiva:** hace referencia a cualquier tipo de conflicto familiar (desavenencias, no relación)	SI	NO
3**. Familia con dificultad para asumir las responsabilidad de atención del paciente:** las dificultades hacen referencia a motivos laborales, cargas familiares, agotamiento y otras limitaciones personales.	SI	NO
4**. Condiciones de higiene personal inadecuadas** **o deficientes:** definido en el mismo enunciado	SI	NO
5.** La vivienda es inadecuada a las necesidades del enfermo:** hace referencia a la existencia de barreras arquitectónicas, falta de espacio, humedades, falta de servicios básicos (agua, luz...)	SI	NO
6.** Se aprecia falta de recursos económicos:** la apreciación hace referencia a la manifestación expresa del enfermo y familia, y también a la impresión de los profesionales (observación)	SI	NO

**Puntuación:** 1 indicador positivo=Riesgo social.
